# Sexual orientation and mental health disparities in US pre-teens: A longitudinal mediation study

**DOI:** 10.1093/eurpub/ckac129.675

**Published:** 2022-10-25

**Authors:** BA Feinstein, A van der Star, KD Dorrell, AJ Blashill

**Affiliations:** 1 Department of Psychology, Rosalind Franklin University of Medicine and Science, North Chicago, IL, USA; 2 Department of Psychology, San Diego State University, San Diego, CA, USA; 3 Joint Doctoral Program in Clinical Psychology, San Diego State University, UC San Diego, San Diego, CA, USA

## Abstract

**Background:**

Sexual minority children are at increased risk for psychopathology compared to their heterosexual peers, but longitudinal studies are needed to determine whether sexual minority identification precedes (rather than co-occurs with) mental health disparities and what may drive these disparities during childhood. The current study examined the longitudinal associations between sexual orientation and mental health over two years in a cohort of U.S. pre-teens with two potential mediators (increased social problems and decreased perceived school safety).

**Methods:**

We used data from Waves 1-3 (2016-2020) of the U.S. Adolescent Brain Cognitive Development study. Multiple linear regression and auto-regressive cross-lagged mediation models were used to examine longitudinal associations and mediation. Analyses accounted for customized sampling weights to correct for attrition and missing data.

**Results:**

The analytic sample included 5,574 children (46.0% assigned female at birth, 55.1% non-Hispanic White). Across waves, beginning to identify as gay/bisexual (0.6-2.7% of sample) was associated with increased internalizing and externalizing problems, and consistently identifying as gay/bisexual (3.4-5.0% of sample) with increased internalizing problems, compared to consistently identifying as heterosexual. For those who consistently identified as gay/bisexual, the widening disparities in internalizing problems were partially explained by increased social problems and decreased perceived school safety.

**Conclusions:**

The health disparities affecting sexual minority children include internalizing and externalizing problems, and are partially explained by increased social problems and decreased perceived school safety. These findings demonstrate that sexual minority identification precedes increases in mental health problems relative to heterosexual children, driven by peer difficulties and feelings of unsafety at school experienced by sexual minority children.

